# Inter- und Transdisziplinarität als normative Dynamik: Herausforderungen und Chancen für die Politikwissenschaft

**DOI:** 10.1007/s11615-023-00453-7

**Published:** 2023-02-23

**Authors:** Antonia Graf, Bastian Loges, Sandra Schwindenhammer

**Affiliations:** 1 Institut für Politikwissenschaft, Westfälische Wilhelms-Universität Münster, Scharnhorststr. 100, 48151 Münster, Deutschland; 2 Institut für Internationale Beziehungen (IIR), Technische Universität Braunschweig, Bienroder Weg 97, 38106 Braunschweig, Deutschland; 3 Institut für Politikwissenschaft, Justus-Liebig-Universität Gießen, Karl-Glöckner-Str. 21E, 35394 Gießen, Deutschland

**Keywords:** Forschungspolitik, Wissensintegration, Nachhaltigkeit, Normenforschung, Teilnehmende Beobachtung, Autoethnografie, Science policy, Knowledge integration, Sustainability, Norm research, Participant observation, Autoethnography

## Abstract

Die Inter- und Transdisziplinarität (ITD) hält seit längerer Zeit Einzug in die Politikwissenschaft und obgleich diese sich regelmäßig mit ihrem Selbstverständnis auseinandersetzt, sind die Konsequenzen von ITD für Forschung und Forschende bislang noch nicht systematisch betrachtet worden. Um Impulse für diese Debatte zu setzen, konzeptualisieren wir ITD als Spektrum zwischen Wissensintegration, Anwendungsbezug und Beteiligung. Wir nutzen die Normenforschung der Internationalen Beziehungen (IB) als theoretischen Referenzrahmen, um die ITD als normative Dynamik zu beschreiben, zu analysieren und zu reflektieren. Autoethnografisch und mittels teilnehmender Beobachtung untersuchen wir ITD als normative Dynamik anhand von drei Forschungsprojekten im Bereich der Nachhaltigkeit. Konkret fragen wir, welche Implikationen die ITD für Forschende und Forschung in der Politikwissenschaft hat. Im Ergebnis zeigt sich, dass ITD sowohl Chancen als auch Herausforderungen bietet. Vor dem Hintergrund der Wissensintegration diskutieren wir die Bedeutung einer an den großen gesellschaftlichen Fragen teilhabenden Politikwissenschaft im Kontrast zu den Präferenzen der ITD für ein bestimmtes Wissens- und Forschungsverständnis. Den Anwendungsbezug der ITD reflektieren wir im Hinblick auf die Möglichkeiten zur Problemlösung und Outputorientierung. Zudem betrachten wir das Teilhabepostulat der ITD und wägen potenzielle demokratisierende Effekte mit den Bedingungen ab, unter denen diese realisiert werden könnten. Abschließend gehen wir darauf ein, wo weitere Forschung sinnvoll erscheint, um die Reflexion über ITD weiter fortzuschreiben.

## Abstract (en)

Inter- and transdisciplinarity (ITD) has been part of political science for quite some time now, but although political science regularly deals with its self-understanding, the consequences for research and researchers of ITD have not yet been systematically considered. To stimulate this debate, we conceptualize ITD as a spectrum of knowledge integration, application, and participation. We use International Relations norm research as a theoretical framework to describe, analyze, and reflect on ITD as a normative dynamic. Autoethnographically and through participatory observation, we examine ITD as a normative dynamic, with insights from three research projects in the field of sustainability. Specifically, we ask what implications ITD has for researchers and research in political science. As a result, we find that ITD offers both opportunities and challenges. In the context of knowledge integration, we discuss the importance of the participation of political science in major societal issues in contrast to ITD’s preferences for a particular understanding of knowledge and research. We reflect on ITD’s application bias in terms of problem-solving opportunities and output orientation. In addition, we consider the participation postulate of ITD and weigh potential democratizing effects against the conditions under which these might be realized. Finally, we address where further research seems useful to continue reflection on ITD.

## Einleitung

Die praktische Anwendbarkeit von Forschungsergebnissen sowie deren Genese ist Dreh- und Angelpunkt aktueller forschungspolitischer Debatten, die Implikationen für die Politikwissenschaft haben. So weist der Koalitionsvertrag an zentraler Stelle auf die „Stärkung von anwendungsorientierter Forschung“ hin, rückt aber zugleich Bürger*innen in den Fokus von Wissenschaftspolitik, indem er „mit Citizen Science und Bürgerwissenschaften Perspektiven aus der Zivilgesellschaft stärker in die Forschung einbeziehen“ (SPD et al. 2021) möchte. In einer Stellungnahme an die zuständige Bundesministerin problematisierte daraufhin die Deutsche Vereinigung für Politikwissenschaft gemeinsam mit vier weiteren sozialwissenschaftlichen Fachverbänden einen zu stark an Anwendung und Transfer ausgerichteten Entwurf zukünftiger Wissenschafts- und Forschungspolitik. Weil sozialwissenschaftliche Forschung „eine essentielle Rolle für den Erhalt und die Gestaltung der Demokratie sowie des gesellschaftlichen Zusammenlebens“ spiele, benötige eine angemessene Erforschung komplexer Thematiken weitere Perspektiven jenseits einer „primär an der Wirtschaft orientierte[n] Verwertungslogik“ (DVPW et al. 2022).

Auch in Ausschreibungsprogrammen von Drittmittelgebern wie der Deutschen Forschungsgemeinschaft (DFG) oder dem Bundesministerium für Bildung und Forschung (BMBF) sind Aspekte wie Anwendungsbezug und Stakeholderorientierung feste Bestandteile. Sie lassen sich als Reaktionen auf komplexe Problemlagen und deren Erforschung und Bearbeitung interpretieren. Das BMBF beispielsweise versteht seine Strategie für Nachhaltigkeitsforschung als explizit inter- und transdisziplinär (BMBF 2020a) und setzt in seinem Rahmenprogramm für die Geistes- und Sozialwissenschaften dezidiert auf Anwendungsbezüge und Bürger*innendialoge (BMBF 2020b). Zudem fördert die DFG neben grundständiger Forschung zunehmend auch Transferprojekte, um „Erkenntnisse der Grundlagenforschung unter Praxisbedingungen zu prüfen oder gemeinsam mit einem Anwendungspartner bis zu einem Prototyp oder einer beispielhaften Anwendung weiterzuentwickeln“ (DFG 2020). Somit wird Inter- und Transdisziplinarität (ITD) von forschungspolitischen Akteur*innen in Deutschland an prominenter Stelle als Instrument thematisiert, das sowohl die wissenschaftliche Basis als auch die notwendige Akzeptanz für künftige Problembearbeitung bereitstellen soll.

In der Literatur gibt es keinen Konsens darüber, wie Inter- und Transdisziplinarität voneinander abzugrenzen seien (Jahn et al. 2021). Grundsätzlich versucht Interdisziplinarität, kognitive und disziplinbezogene Barrieren abzubauen, um über die Integration disziplinär geprägter Expertise zu einer additiven, interaktiven oder gar holistischen Produktion von Wissen zu gelangen (Chaudry und Plischke 2016; Froese et al. 2019). Transdisziplinarität hinterfragt darüber hinausgehend eine generelle Hierarchie zwischen wissenschaftlicher und lebensweltlicher Expertise und setzt auf den gemeinsamen Austausch von Bürger*innen mit Forschenden, Praktiker*innen, Unternehmen und Politik für akzeptable Lösungen, Produkte und/oder Policies (Jooß et al. 2014; Lutz und Bergmann 2018). Beide Forschungsstränge beinhalten geteilte, wenngleich unterschiedlich akzentuierte Charakteristika. Wie wir später genauer ausführen, begreifen wir ITD deshalb als ein Spektrum, das sich zwischen der Integration von Wissen, der Beteiligung unterschiedlicher Akteur*innen sowie der Relevanz von Anwendungsbezügen aufspannt und in dem konkrete Forschungsprojekte erheblich variieren. Somit hat ITD Berührungspunkte zu policynaher und anwendungsorientierter Forschung wie sie in Hochschulen für angewandte Wissenschaften oder Thinktanks lange Tradition hat und ist aufgrund gleichbleibend hoher Drittmittelquoten auch für Universitäten vermehrt ein Thema (DFG 2021).

In diesem Artikel diskutieren wir ITD als normative Dynamik in der Forschungslandschaft, die sowohl Auswirkungen auf die politikwissenschaftliche Forschungspraxis als auch die Rolle der Forschenden hat. Normative Dynamiken sind politische Interaktionen, in denen Normen als Angemessenheitsstandards von diversen Akteur*innen etabliert, verändert, kritisiert oder angepasst werden (Finnemore und Sikkink 1998; Rosert 2022). Als normative Dynamik verändert die ITD solche Angemessenheitsstandards hinsichtlich der Rolle von Wissenschaft und der Bereitstellung von System‑, Orientierungs- und Transformationswissen zur Lösung eines bestimmten Problems. Besonders deutlich wird dies im Bereich der Nachhaltigkeitsforschung: Umweltprobleme mit hoher gesellschaftlicher Relevanz stellen ein komplexes soziotechnisches Gefüge dar und sollen vor dem Hintergrund vielschichtiger politisch-gesellschaftlicher Implikationen gelöst werden (Rittel und Webber 1973), für das Laienwissen unentbehrlich ist (Ansell und Torfing 2021). Grundsätzlich verweist nachhaltiges Handeln auf eine ökologische, ökonomische und soziale Dimension, wobei Nachhaltigkeitspolitiken entlang dieser Dimensionen variieren (Davies 2013) und Strategien, Handlungsprogramme und Adaptionslogiken zwischen Effizienz, Konsistenz und Suffizienz informieren. Zugleich bringen Lösungen wegen ihrer potenziellen Eingriffstiefe in den gesellschaftlichen Alltag möglicherweise Akzeptanzprobleme mit sich. Deshalb können und sollen Analyse, Erarbeitung und Umsetzung von Lösungskonzepten nicht ohne die Beteiligung derer bewerkstelligt werden, die von Problemen betroffen sind oder von Entwicklungen profitieren (Michelsen und Adomßent 2014). ITD-Forschung fördert zudem den jüngst von Hickmann et al. (2022, S. 154) angemahnten Diskurs über Nachhaltigkeit quer durch alle Wissenschaften, bei dem insbesondere die Politikwissenschaft eine stärkere interdisziplinäre Auseinandersetzung mit fachwissenschaftlichen Arbeiten anderer Disziplinen suchen solle. Letztlich bleibt diese Mahnung nicht auf Umweltprobleme beschränkt. Die COVID-19-Pandemie, Fragen nach Energiesicherheit oder atomarer Rüstungskontrolle zeigen, dass politische Probleme zunehmend inter-, wenn nicht sogar transdisziplinär thematisiert werden (müssen), um angemessene Problemlösungen zu ermöglichen.

Auch in Debatten über die Selbstverortung, Selbstvergewisserung und Weiterentwicklung der Politikwissenschaft (Schöne und Bergem 2022; Fröhlich et al. 2017) werden der Wissens- und Praxistransfer von Forschungsergebnissen, neue Formen der öffentlichen, politischen und transnationalen Wissenschaftskommunikation sowie die Rolle der Disziplin als öffentliche Wissenschaft für gesellschaftliche Problemlösung bereits diskutiert. Letzteres impliziert einerseits die Reflexion über den gesellschaftlich-politischen Einfluss der Politikwissenschaft zwischen Information, Beratung und Legitimation sowie das Potenzial von Bürger*innenbeteiligung in partizipativen Formaten der Politikberatung (Glaab 2022). Andererseits will die Politikwissenschaft ausloten, wie „offenere und alternative Wege des Zugangs zu Politik und Gesellschaft“ (Hegemann und Niemann 2022, S. 244) beschritten und der klassische Wissenstransfer durch neue Formen des Wissensaustauschs mit der Gesellschaft in partizipativen Forschungsformaten ergänzt oder gar ersetzt werden können (Hegemann und Niemann 2022, S. 240).

Im Folgenden zielen wir auf der Basis von drei inter- und transdisziplinären Forschungsprojekten im Nachhaltigkeitsbereich auf eine methodisch geleitete Reflexion von ITD in der Politikwissenschaft aus Sicht der IB-Normenforschung. Das Papier fragt:* Welche Implikationen hat die Verbreitung von ITD als normative Dynamik für Forschung und Forschende in der Politikwissenschaft?* Für die empirische Analyse nutzen wir das Instrumentarium der teilnehmenden Beobachtung, um unsere Erfahrungen zu protokollieren, zu systematisieren und vergleichend auszuwerten. Die Normenforschung interessiert sich für die „Wirkung von Normen sowie ihre Entstehung und Diffusion“ (Rosert 2022, S. 2). Sie eignet sich zur Analyse und Reflexion von Herausforderungen und Chancen von ITD, weil sie für die kritische Auseinandersetzung mit normativen Dynamiken verschiedene Konzepte und analytische Begriffe bereitstellt, die eine Sichtbarmachung normativer Einschreibungen des Forschungsgegenstands wie des Forschungsprozesses ermöglicht. Normen werden in der Normenforschung stets im Wechselspiel zwischen Akteur*in und Struktur gedacht, womit die Perspektive den prozeduralen Charakter des Normativen unterstreicht und deshalb besonders geeignet ist, normative Veränderungen für Handeln sowie Ordnungsbildung auszuweisen (Glaab et al. 2021; Linsenmaier et al. 2021). Unsere Ergebnisse schließen dabei auch an Diskussionen zum Wandel der politischen Relevanz von Expertise an (Bogner 2021; Büttner und Laux 2021), wobei wir aber die normativen Auswirkungen der ITD auf die Disziplin und die Forschungscommunity in den Vordergrund stellen.

Wir argumentieren, dass ITD spezifische Implikationen für die Rolle und Agency von Forschenden und damit verbundene Machteffekte hat, die nicht nur in der Hervorbringung, sondern auch in der Disziplinierung und Naturalisierung von Wissen und Praktiken liegen. Weil ITD nicht nur Wissen produzieren oder unterschiedliche Wissensbestände integrieren, sondern zugleich politische Legitimität und Akzeptanz herstellen möchte, werden Forschende potenziell zu politisch Intervenierenden, was eine Reflexion von (Macht‑)Beziehungen innerhalb des Forschungsprozesses erfordert. Dazu stellen wir zunächst das Leitbild von ITD als transformativer Wissenschaft vor, verorten unseren Zugang in der IB-Normenforschung sowie der teilnehmenden Beobachtung und beschreiben, analysieren sowie reflektieren unsere Erfahrungen in drei ITD-Nachhaltigkeitsprojekten. Daran anschließend diskutieren wir die Implikationen von Wissensintegration, Anwendungsorientierung und Beteiligung, die wir als Herausforderungen, aber zugleich auch als Chancen für die Politikwissenschaft verstehen. Schließlich regen wir weitere politikwissenschaftliche Auseinandersetzung mit ITD an, die über die IB-Normenforschung hinausgeht.

## Inter- und Transdisziplinarität als Spektrum und normative Dynamik

Ohne die Unterschiede zwischen Inter- und Transdisziplinarität leugnen zu wollen, diskutieren wir beides im Folgenden als ITD gemeinsam, weil eine strikte Trennung weder der Komplexität noch der Diversität empirisch beobachtbarer Akteurskonstellationen, Wissensproduktion und Wissensanwendung gerecht wird. Anstatt eine Dualität von transdisziplinärer und nichttransdisziplinärer Forschung zu unterstellen (siehe auch die Problematisierung bei Jahn et al. 2021), Forschungsfragen entweder der gesellschaftlichen oder der wissenschaftlichen Domäne zuzuweisen oder die Identität von Akteur*innen als praxis- oder forschungsnah zu diskutieren, schlagen wir ein Verständnis von ITD als normativer Dynamik auf einem Spektrum vor, das sich zwischen der Integration von Wissen, der Beteiligung unterschiedlicher Akteur*innen sowie der Relevanz von Anwendungsbezügen aufspannt. Innerhalb des Spektrums von ITD-Forschung können konkrete Forschungsprojekte erheblich variieren.

### ITD als Spektrum transformativer Wissenschaft

Für ITD-Forschung ist zunächst die Integration von Wissen zentral, damit es zur Lösung eines Problems herangezogen werden kann. Spätestens seit Mitte der 1990er-Jahre werden unterschiedliche Modi der Wissensproduktion außerhalb der Universität (kritisch) im akademischen Umfeld besprochen (Gibbons et al. 1994). Basierend auf dem „multilevel multigoal education/innovation system“ des Physikers Erich Jantsch (1972, S. 14) unterscheidet Becker (2002, S. 7) drei Diskurs- und damit Wissensarten, die als Fragen an sozial-ökologische Probleme gerichtet werden können, um sie für Forschung innerhalb des ITD-Spektrums zu übersetzen. Während die deskriptive Frage „Was ist der Fall?“ zur Etablierung von Systemwissen führt, resultiert die normative Frage „Was sollen wir tun?“ in Orientierungswissen. Die daran anschließende operative Frage „Was können wir tun?“ zielt auf die Bereitstellung von Transformationswissen ab (siehe bspw. auch die Übersicht bei Jahn et al. 2012 in Anlehnung an Becker). Je nach ITD-Forschungsprojekt können verschiedene Wissensarten unterschiedlich akzentuiert sein. Gemein ist Projekten in der ITD aber, dass sie auf eine Synthese von unterschiedlichem Wissen und dessen Begriffen zielen (Brunnengräber und Smeddinck 2016; Feldhoff et al. 2019).

Im ITD-Forschungsprozess können neben Wissenschaftler*innen diverser Disziplinen in variierendem Ausmaß auch Praktiker*innen oder Bürger*innen in alle Phasen des Forschungsprozesses einbezogen sein (Jahn et al. 2012; Schneidewind und Singer-Brodowski 2015), wodurch verschiedene Wissensarten mit „Praxisakteure[n] permanent verschränkt und rückgekoppelt werden“ (Schneidewind und Singer-Brodowski 2015, S. 18). Dazu implementiert ITD-Forschung vielfältige und experimentelle Beteiligungsformate wie Bürger*innendialoge, Ko-Kreation oder Reallabore. Durch die Beteiligung unterschiedlicher Akteur*innen soll die Interaktion mehrerer Disziplinen einen produktiven, fremden Blick auch auf die eigene Fachkultur erzeugen und durch die Irritation disziplinärer Traditionen und Wissensbestände eine zusätzliche epistemische Funktion erfüllen (Wissenschaftsrat 2020, S. 15). Dabei ist ITD-Forschung auf ein geteiltes Verständnis grundlegender Begriffe der beteiligten Akteur*innen angewiesen, welches gemeinsame Fragestellungen erst ermöglicht. Zugleich erfordert ITD-Forschung von beteiligten Akteur*innen die Grundbereitschaft zum Dialog und zur Reflexion des eigenen Wissenschaftsverständnisses. Allerdings ergeben sich hier spezifische Problemlagen: Je größer und heterogener der Kreis von Beteiligten in ITD-Forschungsprozessen ist, desto manifester stellt sich möglicherweise die Herausforderung dar, Verständigung und Dialog zu organisieren (Godemann und Michelsen 2008).

Innerhalb der ITD ist Forschung jenseits von Wissensproduzentin auch gesellschaftliche Problemlöserin, weil sie Phänomene nicht nur verstehen, sondern auch aktiv gestalten und zur Transformation beitragen möchte (Vilsmaier und Lang 2014, S. 97). Der Anwendungsbezug von ITD-Forschung ergibt sich unmittelbar, wenn verschiedene Wissensformen direkt an die handelnden Akteur*innen zurückgespiegelt werden und so auch das untersuchte System verändern (Schneidewind und Singer-Brodowski 2015, S. 15). Nicht selten mündet ITD-Forschung, die einen starken Fokus auf Transformationswissen legt, direkt in politikrelevante Handlungsempfehlungen, die von maßgeblichen Akteur*innen aufgegriffen werden (sollen). Gleichzeitig kann aber auch jene ITD-Forschung, die a priori keinen starken Fokus auf Transformationswissen legt, einen bedeutsamen Anwendungsbezug erhalten, nämlich dann, wenn Forschungsergebnisse quasi nichtintendiert breit gesellschaftspolitisch rezipiert werden.

### Wissenschaftspolitische Akteur*innen und die normative Dynamik der ITD

Inwiefern kann ITD als normative Dynamik in der Wissenschaftspolitik gelesen werden? Im Folgenden argumentieren wir, dass sich mit der ITD in der Forschungslandschaft beobachtbarer Wandel vollzieht, also Dynamiken sichtbar werden, die Implikationen für die Politikwissenschaft haben. Normen gelten uns als kollektive Standards angemessenen Verhaltens, durch die soziales Handeln von Akteur*innen in gesellschaftliche Kontexte eingebettet wird (Finnemore und Sikkink 1998). Dabei initiieren und perpetuieren sie individuelle wie kollektive Sinnstiftungsprozesse, sind aber zugleich grundsätzlich umstritten und können politischen Konsens ebenso repräsentieren wie Kontestation (Wiener 2018). Somit sind normative Dynamiken immer politisch und zielen auf die wechselseitige Konstitution von Akteur*in und Struktur, die der ITD sowohl disziplinäres als auch gesamtgesellschaftliches Veränderungspotenzial zuschreibt. Wenn ITD als Instrument der Transformation begriffen wird, mit dessen Hilfe konkrete Handlungsempfehlungen formuliert werden, um legitime und effektive Lösungen für gesellschaftliche Probleme bereitzustellen, kann der ITD also eine Normsetzungsabsicht unterstellt werden. Die beobachtbare Dynamik verläuft dabei in unterschiedliche Richtungen und lässt sich an diversen Akteur*innen feststellen: Aus Richtung der geldgebenden Institutionen erfolgt die (häufig explizite) Aufforderung an Wissenschaftler*innen, ITD in die Forschung zu integrieren. Sie sollen nicht nur Resultate erarbeiten, sondern diese auch für politische Akteur*innen in eindeutige Vorschläge in Form von Handlungsempfehlungen übersetzen.

Zugleich zeigt sich im Nachhaltigkeitsbereich ein neues Protestpotenzial (Fridays for Future, Letzte Generation, Extinction Rebellion und andere) verbunden mit einem Moment der kritischen Öffentlichkeit, das von gestiegenen gesellschaftlichen Erwartungen mancher Bürger*innen an demokratische Beteiligung zeugt. In diesem Zusammenhang diffundiert ITD über Zukunftsdialoge, Reallabore, Bürger*innenräte und Stakeholderdialoge, die inkrementellen, ko-kreativen gesellschaftlichen Wandel in Aussicht stellen, in Forschungseinrichtungen, NGOs, Verbände, Stadtverwaltungen und Ministerien. In Summe: Erwartungen und Ansprüche von unterschiedlichen Akteur*innen an Forschung und ihre Praktiken induzieren normative Dynamiken, bei denen jenseits der spezifischen Fragestellung und zu erbringender Analyse auch Teilhabe, Problemlösung und Beratung diskutiert werden.

Allerdings wird die Zunahme von ITD nicht durchgängig positiv bewertet, was normative Dynamiken erneut unterstreicht: Dem Wissenschaftsrat folgend (2015, S. 27) sehen Kritiker*innen durch eine Verlagerung des Referenzrahmens wissenschaftlicher Erkenntnisgewinnung die Autonomie der Hochschulen und die Freiheit der Wissenschaft sowie die epistemischen Eigengesetzlichkeiten von Wissenschaft gefährdet. ITD gehe mit einer Abwertung der Disziplinarität einher, weshalb eine Verflachung der Forschung drohe (Loenhoff 2021). Auch der Wissenschaftsrat misst den Disziplinen große „Bedeutung als Ordnungsstrukturen des Wissenschaftssystems“ (Wissenschaftsrat 2020, S. 7, Fußnote 1) bei, wohingegen eine „systemische Funktion von Interdisziplinarität“ darin liegt, „die bestehende disziplinäre Ordnungsstruktur in Frage zu stellen und dynamisch fortzuschreiben“ (Wissenschaftsrat 2020, S. 9).

## Kritische IB-Normenforschung als autoethnografische Beobachtung

Im Folgenden führen wir die IB-Normenforschung als theoretischen Referenzrahmen ein, der nicht nur Phänomene beschreiben und analysieren kann, sondern sich zugleich kritisch mit normativen Einschreibungen und Entwicklungen auseinandersetzt. Zunächst charakterisieren wir die kritische IB-Normenforschung als Reflexionsinstrument und gehen dann auf die teilnehmende Beobachtung und die Autoethnografie als Erhebungsmethoden ein, mittels derer wir unsere ITD-Erfahrungen rekonstruktiv erfassen, systematisieren und Schlussfolgerungen formulieren.

### Kritische IB-Normenforschung als Instrument der Beobachtung, Analyse und Reflexion

Die IB-Normenforschung, die sich mit spezifischen Normen und den aus ihnen erwachsenden Dynamiken beschäftigt (Rosert 2022), lässt sich zur Bestimmung und Reflexion der Implikationen von ITD als normative Dynamik für politikwissenschaftliche Forschung und Forschende gleich dreifach nutzen: (i) Sie kann Wandlungsprozesse verdeutlichen, indem sie spezifische Begriffe zu deren Beschreibung vorhält (Graf et al. 2021; Jurkovich 2020). (ii) Zudem vermag sie normative Dynamiken mit Verweis auf Strukturbildung, Prozesse und Akteur*innen zu analysieren (Lantis 2017; Sandholtz 2017). (iii) Schließlich begreift sie sich als Reflexionsinstrument, das über einen poststrukturalistisch geprägten, diskursiven Machtbegriff die Machteinschreibungen von normativen Ordnungen und die Situiertheiten innerhalb dieser Ordnungen erfassen kann (Epstein 2012).

(i) Zur Beschreibung normativen Wandels stellt die IB-Normenforschung ein Begriffsinstrumentarium bereit, das zur Definition von Normen auf intersubjektive Angemessenheitsstandards, kollektive Erwartungen sowie angemessenes Verhalten auf Basis einer gegebenen Identität verweist (Finnemore und Sikkink 1998, S. 891). Konkret adressieren sie Normativität in Ge- wie Verboten und fokussieren kollektiv erwartetes Verhalten, das sich in regelmäßiger (diskursiver) Praxis als Normalität zeigt. Ein solcher Stabilitätsbezug wird aktuell hinterfragt, weil Normen zunehmend prozedural als Interaktion gedacht und ihnen kein intersubjektiv geteiltes Verständnis von Angemessenheit unterstellt werden kann (Linsenmaier et al. 2021). Diese Hinterfragung eines normativen *shared understandings* über das *meaning-in-use* gilt dabei als Kontestation, die zu variierenden Ergebnissen für die betreffenden Normen führt, weil sie die Robustheit wie Resilienz von Normen adressiert (Deitelhoff und Zimmermann 2019; Lantis und Wunderlich 2022) und zugleich thematisiert, wie globale Normdynamiken über die Beteiligung von Betroffenen Legitimität herstellen können (Acharya 2004; Wiener 2018).
(ii) Als Analyseinstrument will die IB-Normenforschung normative Dynamiken in ihrer Varianz verstehen und erklären (Betts und Orchard 2014; Deitelhoff 2006; Loges 2013). Weil Normen dynamische Einheiten darstellen, die sich nicht linear über verschiedene Ebenen oder Kontexte hinweg verbreiten, werden sie auf ihrer „Reise“ (Zwingel 2012) durch Prozesse der Lokalisierung und Translation geformt: Lokale, religiöse, kulturelle oder ökonomische Kontexte, Skripte und Praktiken lösen Irritationen, Diskrepanzen oder Feedbackschleifen zwischen lokaler und globaler Ebene aus und führen zu Reformulierungen, Adaptionen oder auch Ablehnung von Normen (Acharya 2004; Graf 2016; Schwindenhammer 2018; Zimmermann 2017). Besondere Relevanz kommt dabei sogenannten Normunternehmer*innen zu, die eine bestehende Norm hinterfragen, normativen Wandel anstoßen und versuchen, weitere Akteur*innen als Adressat*innen eines neuen Vorschlags zu überzeugen. Über eine Mischung von identitär wirkenden Überzeugungsprozessen (Deitelhoff 2006, S. 14) und sozialem Druck oder Sanktionen (Axelrod 1986, S. 1097) zielen sie darauf ab, diese neue Vorstellung diskursiv wie praktisch zu verankern. Allerdings wird angesichts empirischer Trends zunehmend eine Dichotomie von *Normmakern* und *Normtakern* hinterfragt. Aktuelle IB-Normenforschung unterstreicht deshalb, dass auch Normadressat*innen eigenständige Agency zukommt (Acharya 2004) und sich auch sogenannte *Antipreneure* ähnlicher Überzeugungspraktiken bedienen, um den Status quo beizubehalten bzw. gegen neue normative Dynamiken zu resistieren (Bloomfield 2016).
(iii) Schließlich nutzen wir die IB-Normenforschung als Reflexionsinstrument, das Machtbeziehungen innerhalb des Forschungsprozesses hinterfragt (Glaab et al. 2021). Ausgehend von interpretativen oder dekonstruierenden Verfahren kritisiert die IB-Normenforschung einen liberalen Bias und die daraus folgende Machtblindheit von Forschung. Macht zeigt sich demnach in einer Normalisierung bestimmter Vorstellungen, Praktiken und Wissensbestände, maßgeblich jener des Globalen Nordens (Epstein 2012; Zarakol 2014), die zu einer Naturalisierung von Normen beiträgt (Engelkamp et al. 2012; MacKenzie und Sesay 2012). Teil dieser Kritik an der Machtblindheit ist die fehlende Auseinandersetzung mit Situiertheit, also mit der Verortung Forschender innerhalb der Prozesse, die sie analysieren und dadurch selbst mit beeinflussen. Dementsprechend inkludiert IB-Normenforschung eine Reflexion der eigenen Normativität und Rolle innerhalb von Forschungsprozessen (Inayatullah und Blaney 2012).

Obgleich die kritische IB-Normenforschung innerhalb unserer Projekte nicht zentral ist, sind wir als Forschende in der Normenforschung sozialisiert und haben an anderer Stelle unsere Projektgegenstände bereits normtheoretisch analysiert (Loges und Jakobi 2020; Schwindenhammer 2017; 2018; Stockmann und Graf 2019; 2020, 2022). Unsere gemeinsame Perspektive auf normative Dynamiken half aber nicht nur, die individuellen Erfahrungen besser artikulieren und kommunizieren zu können, sondern auch, die Implikationen der ITD als normative Dynamik in der Politikwissenschaft im Austausch zwischen den Autor*innen systematisch zu erfassen und zu ordnen.

### Teilnehmende Beobachtung und Autoethnografie als Erhebungsmethoden

Als Politikwissenschaftler*innen mit ITD-Projekten rekonstruieren wir eine normtheoretisch geprägte Zusammenschau ähnlicher Erfahrungen. Dazu nutzen wir einen breiten ethnografischen Zugang (Birkholz et al. 2020; Vrasti 2017), der unsere ITD-Projekte als Feld versteht, in dem wir uns bewegen und welches wir mittels teilnehmender Beobachtung, aber auch durch autoethnografische Reflexion betrachten.

Teilnehmende Beobachtung geht von der Überlegung aus, dass sich durch räumliche Nähe auch spezifische Möglichkeiten zur analytischen Durchdringung von Situationen ergeben (Lüders 2006). Durch die Gleichzeitigkeit von teilnehmender Nähe und beobachtender Distanz ergibt sich methodisch wie praktisch allerdings ein Widerspruch (Beer 2015; Kromrey et al. 2016). Die Vorschläge der Ethnografie zur Bearbeitung dieses Spannungsverhältnisses oszillieren zwischen bewusster Vergrößerung der Nähe als Verdichtung der Beschreibung (Klotz und Lynch 2007) und der Erhöhung von Distanz über eine künstliche Befremdung der Beobachter*innen (Hitzler 2006). In der expliziten Reflexion der Differenz von Nähe und Distanz, von Beobachtung und Teilnahme wird eine Möglichkeit zur kritischen Auseinandersetzung mit sozialer Praxis gesehen (Vrasti 2008). Somit ist teilnehmende Beobachtung stets gleichermaßen die Beobachtung, Interpretation und Analyse von Situationen, was auch die generelle Situiertheit von Forschenden unterstreicht (Halperin und Heath 2012). Dies wird über Feldnotizen, also das Protokollieren der Interaktion, oder Forschungstagebücher geleistet, die mit den praktischen Erfahrungen und Erinnerungen der Forschenden gespiegelt werden (Schwartz-Shea und Yanow 2012).

Wir haben unsere teilnehmenden Beobachtungen in den ITD-Projekten über umfangreiche Notizen und Protokolle dokumentiert und deren Ergebnisse anschließend vergleichend systematisiert. Hierbei ist der Hinweis wichtig, dass wir nicht die der Interaktion zugrunde liegenden Inhalte oder Gegenstände dokumentieren. Der Fokus des Beobachtens und Protokollierens lag nicht auf Fragen, Interessen oder Produkten einzelner Projektpartner*innen, sondern auf Prozessen der inter- wie transdisziplinären Interaktion. Unsere Beobachtungen haben wir um eine autoethnografische Dimension ergänzt, um unsere Erfahrungen sowie die eigene Situiertheit zu reflektieren und sich wandelnde Normalitäten wie Normativitäten empirisch fassen zu können (Adams et al. 2020; Neumann 2010). Dabei schließen wir an die Nutzung von (Auto‑)Ethnografie in den IB zur Reflexion an (Brigg und Bleiker 2010; Vrasti 2008): Autoethnografie macht die Position von Forschenden samt ihren Erfahrungen explizit und ermöglicht dadurch transparente Reflexion. So ergänzt die Autoethnografie die Normenforschung in methodischer Hinsicht. Konkret haben wir zunächst individuell aus den Projekten kürzere Texte formuliert, Mindmaps entwickelt und Differenzen innerhalb der ITD-Interaktionen offengelegt. Ein digitales Whiteboard hat die Zusammenarbeit ermöglicht und gleichzeitig als Archiv gedient. Dieses Vorgehen versetzte uns in die Lage, ähnliche und abweichende Aspekte zu identifizieren und unsere Beobachtungen in Relation zueinander zu setzen (Breidenstein et al. 2020). Auf diese Weise ist eine Matrix zur ITD-Forschung entstanden, welche die Beschreibungs‑, Analyse- und Reflexionsmöglichkeiten der IB-Normenforschung nutzt und sie zu nachfolgenden Einsichten über ITD-Forschung und -Forschende systematisch ausbaut.

## ITD und normative Dynamiken: Forschung und Forschende in drei ITD-Nachhaltigkeitsprojekten

Im Folgenden legen wir zur Beantwortung der übergreifenden Frage, welche Implikationen die ITD als normative Dynamik für Forschung und Forschende in der Politikwissenschaft hat, Erkenntnisse aus drei Verbundforschungsprojekten dar, in denen wir in den letzten Jahren praktische ITD-Erfahrungen gesammelt haben. Unsere drei Projekte verorten sich in der drittmittelgeförderten Nachhaltigkeitsforschung, in der zu ITD die breit akzeptierte Auffassung besteht, dass komplexe Ursache-Wirkungs-Beziehungen schwer zu überblicken sind und zur Problemlösung die Expertise verschiedener Disziplinen benötigt wird (Lang et al. 2012). Thematisch beschäftigen sich die Projekte mit der Vermeidung von Plastikverschmutzung in Abwässern, nachhaltiger Mobilität in urbanen Räumen und kreislaufbasierter Nahrungsmittelkultivierung in Metropolregionen.

Dass es sich bei unseren ITD-Projekten nicht um Einzelphänomene handelt, verdeutlicht der Kontext, in dem sie angesiedelt sind. Alle drei sind Teile von großen, längerfristig angelegten BMBF-Förderlinien („Sozial-ökologische Forschung“, „Agrarsysteme der Zukunft“ und „Plastik in der Umwelt“), in denen die Bekanntmachungen ähnliche Ziele hinsichtlich Beteiligung, Wissensintegration und Anwendungsbezug von Forschungsergebnissen vorgeben: Es sollen Problemlösungen „durch eine neuartige Qualität des Dialogs und der Zusammenarbeit in inter- bzw. transdisziplinären Kooperationen und Netzwerken“ (BMBF 2016a) und „unter Einbezug von Anwendern arbeitsteilig und partnerschaftlich“ erarbeitet werden (BMBF 2016b). Im Forschungsprozess sollen Wissens- und Forschungsbedarf „von den verschiedenen Akteuren über wissenschaftliche Disziplin- und Systemgrenzen hinweg schrittweise erhoben, zusammengeführt, integriert und zu Innovationen umgesetzt werden“ (BMBF 2016a). Denn um „in der Praxis umsetzbare Lösungswege erarbeiten zu können, ist es unerlässlich, dass Akteure aus Zivilgesellschaft und Wirtschaft in den Forschungsprojekten zumindest mitwirken, wenn nicht sogar diese initiieren und federführend vorantreiben“ (BMBF 2015, S. 11). Letztlich soll so „Wissen […] schneller für Entscheidungsträger in Wirtschaft, Politik, Verwaltung und Zivilgesellschaft verfügbar“ gemacht (BMBF 2016b) beziehungsweise „durch neue, innovative Produktionsmethoden und -formen nachhaltige und effiziente Lösungsmöglichkeiten“ für die technologische Bearbeitung entwickelt werden (BMBF 2016a).

In den drei Projekten geht es um strittige Fragen, wie normative, technische und politische Verhaltenserwartungen so entwickelt und formuliert werden können, dass sie weitgehende Akzeptanz und damit Legitimität entfalten können. Jedoch ist die ITD-Ausprägung innerhalb der drei Projekte unterschiedlich, obgleich alle explizit inter- wie transdisziplinär arbeiten und jenseits von Systemwissen, auch zentral auf Orientierungs- und Transformationswissen abzielen: Während zwei Projekte gemeinsam mit Praxispartner*innen Produkte testen oder Verfahrensansätze entwickeln, die auch patentrechtlich relevant sind, haben die Praxispartner*innen im dritten Projekt eine feedback- und impulsgebende Funktion in der Entwicklung von Fragestellungen bis hin zur Überprüfung von Ergebnissen. Hinzu tritt ein variierendes Bewusstsein für ITD und dessen Reflexion innerhalb der Projekte, welches – wenn auch nur implizit – im Sinne eines reziproken Verhältnisses wiederum auf das jeweilige Verständnis von Nachhaltigkeit im Projekt wirkt. Somit unterscheiden sie sich eher in Bezug auf Beteiligung und die Instrumente der Wissensintegration, nicht aber hinsichtlich der vorzulegenden Wissensarten.

Im Folgenden gehen wir zunächst auf die zentrale Beobachtung einer Pluralisierung von Angemessenheitsstandards in unseren ITD-Nachhaltigkeitsprojekten ein. Anschließend analysieren wir die Wirkung der Normdynamiken auf unterschiedliche Kontestationsprozesse und fragen nach relevantem Wissen sowie veränderten Rollen für Forschende. Schließlich nutzen wir die angestellten normtheoretischen Überlegungen, um eine Reflexion von Wandel und Praktiken zu initiieren, die sich vor allem auf die Situiertheit von Forschenden und die Machteinschreibungen von ITD-Forschung bezieht.

### Beschreibung: Pluralisierung von Angemessenheitsstandards

Innerhalb der Projekte beobachten wir die Pluralisierung von Angemessenheitsstandards als eine zentrale Entwicklungsdynamik, die einerseits aus den inhaltlichen Bezügen und Forschungsgegenständen sowie andererseits aus dem Prozess des Forschens selbst resultieren. Solche Dynamiken setzen Normen in neue Relationen, z. B. Nachhaltigkeit und ITD, was zugleich mit dem mehrdeutigen Nachhaltigkeitsbegriff in der Nachhaltigkeitsforschung (Renn et al. 2007; Wichterich 2012) korrespondiert, dessen Umstrittenheit zu einer paradigmatischen Pluralisierung verschiedenster Deutungen sowie daraus abgeleiteten Transformationspfaden und Ansätzen führt (Breitmeier et al. 2021b, a; Henkel et al. 2018).

Jenseits nachhaltigkeitsorientierter Problemdefinitionen und -lösungsvorschlägen werden in unseren Projekten Vorstellungen von disziplinärer und ITD-Forschung sichtbar. Die verschiedenen Disziplinen und Praxispartner*innen aus Verbänden, Verwaltung, Nichtregierungsorganisationen oder Unternehmen bringen ihre spezifischen teils unterschiedlichen Normen ein. Zugleich gilt ITD-Forschung akteur*innen- und disziplinenübergreifend prinzipiell als erfolgsversprechender im Gegensatz zu Forschung, die nicht zentral auf Problem- und Lösungsorientierung fokussiert. Darüber hinaus erweist sich die Programmatik des Fördermittelgebers als einflussreich, die im Bereich der Nachhaltigkeit oder in der Forschungspolitik als normative Setzung gelesen werden kann: Obgleich in der Forschungspraxis noch Unklarheit oder Uneinheitlichkeit über die Problemdefinition und ihre praktischen wie normativen Implikationen herrscht, erscheinen sie in den jeweiligen Förderlinien und Forschungsanträgen bereits definiert. Mit dieser Situation gehen unsere Projekte recht unterschiedlich um: Während sich in einem Projekt die Projektpartner*innen in mehreren inter- und transdisziplinären Fortbildungen mit dem Nachhaltigkeitsverständnis im Projekt auseinandergesetzt und im Bereich Orientierungswissen bewusst für eine starke Nachhaltigkeit entschieden haben, gab es bei den anderen Projekten keine explizite Auseinandersetzung darüber.

Insgesamt legen unsere Beobachtungen nahe, dass sich Normativität in den Projekten mit einer ITD-Herangehensweise und dem darin quasi vorgeschriebenen Dialog zunächst pluralisiert. Dabei entstehen komplexe Dynamiken, die sich nur bedingt über *shared understandings* stabilisieren lassen. Allerdings stellen wir fest, dass innerhalb der Projekte die normativen Fragen nach Umwelt- oder Gesundheitsschutz, konkret etwa Wasserreinheit, Plastikvermeidung oder Schadstoffbelastung in der Atemluft, nach und nach in spezifischen Terminologien diskutiert werden: Je komplexer die Diskussion wird, umso schwieriger erscheint die inter- und transdisziplinäre Verständigung. Im Hinblick auf den Forschungsoutput erhalten angesichts dieser Komplexität Machbarkeits- und Verwertungslogiken viel Raum und können multidimensionale Forschungsfragen zugunsten gut kommunizierbarer Ergebnisse verdrängen. Zugleich werden Vorschläge stets vor dem Hintergrund einer Implementationsperspektive diskutiert, die Dritte und die Kommunikation mit ihnen betreffen, womit die Nähe zur Anwendung in der Praxis immer zu einem Aspekt der Forschung selbst wird. Themen wie die marktförmige Gestaltung von Produkten und die technische Lösbarkeit von Problemen erscheinen dadurch attraktiver und fordern damit gleichzeitig die Angemessenheitsstandards anderer Projektpartner*innen heraus.

### Analyse: Neue Normdynamiken, Kontestation von Wissen und verstecktes Normunternehmer*innentum

Normtheoretisch gewendet zeigt die Pluralisierung von Angemessenheitsstandards normative Dynamiken an, die sich ergeben, wenn unterschiedliche Akteur*innen nicht nur Wissen integrieren wollen, sondern neben deskriptivem System-, auch normatives Orientierungswissen und anwendungsbezogenes Transformationswissen bereitstellen sollen. Bereits die Etablierung von Systemwissen kennzeichnet überlappende Lokalisierungs‑, Translations- und Kontestationsprozesse durch variierende Praktiken: Weil die Probleme mit Agrarsystemen, Mobilität oder Plastikverschmutzung disziplinär unterschiedlich wahrgenommen und bewertet werden, stehen die Projekte vor der Herausforderung, innerhalb der beteiligten Disziplinen und Wissenschaftler*innen gemeinsame Begriffe und Problembeschreibungen vorzulegen, auf die sich geeinigt werden kann. Dies wird in der Praxis umgesetzt, indem bei der Entwicklung von gemeinsamen Glossaren oder auf interkonsortialen Workshops Problembeschreibungen oft so *lokalisiert *werden, dass sie anschlussfähig an die am Forschungsprojekt beteiligten Disziplinen sind. Im Ergebnis kommt es dabei bereits bei Systemwissen zu einer Verengung auf Teilaspekte oder zu einer Priorisierung bestimmter Problembereiche.

Ist die (Nicht‑)Integration von disziplinärem Wissen im interdisziplinären Austausch gleichermaßen zentral wie umstritten, kommen im transdisziplinären Austausch noch weitere Wissensformen wie Alltags- oder Professionswissen hinzu, wobei aller Offenheit zum Trotz auch Wissen exkludiert werden kann, wenn es als nicht problemlösungsrelevant im Sinne der Projektziele wahrgenommen wird. So entstehen Kontestationsprozesse, die die Robustheit von Normen und ihrer Legitimität berühren. Bemerkenswert ist, dass sich die Kontestation nicht allein auf normative Ansprüche etwa von Nachhaltigkeitskonzeptionen bezieht. Stattdessen verweist sie projektintern auch auf die normativen Einschreibungen wissenschaftstheoretischer Logiken: Sie verdeutlichen die forschungspraktische Konkurrenz von positivistisch erlangtem Wissen mit seiner faktenbasierten Messbarkeit und Indikatorenorientierung, z. B. in der Risikobewertung und anderem, postpositivistisch erhobenem Wissen, das beizeiten den projektbezogenen Problemlösungsstrategien entgegenstehen mag. In ITD-Projekten trifft also unterschiedliches Wissen aufeinander und muss kalibriert werden: So werden bestimmte Wissensbestände und ihr Zustandekommen, also die Expertise, explizit als Grundlage von Kontestation genutzt und zugleich hinterfragt. Während in der ITD-Praxis die Auseinandersetzung mit Fragen von Wissen, Wissensintegration und auch dem politischen Potenzial von Wissen andauernd stattfindet, ist die Rolle von Wissen in der IB-Normenforschung, insbesondere seine Funktionen für Normentstehung und Kontestationsprozesse, noch weitgehend ungeklärt (Breitmeier et al. 2021a; Haas 2019; Hansen-Magnusson et al. 2020; Peterson 2019).

Weitere Effekte aus der ITD für den Forschungsprozess werden bei der Erarbeitung disziplinübergreifender Handlungsempfehlungen deutlich, die Probleme in einem normativ gewünschten Sinne lösen und somit Orientierungswissen bereitstellen sollen. Auch hier wirken disziplinäre Unterschiede, etwa indem auf stärkere Regulierung, technologische Innovation, Umweltschutz oder nachhaltigen Konsum gesetzt wird. Solche diskursiven Auseinandersetzungen über disziplinäre wie lebensweltliche Expertise vor dem Hintergrund der gemeinsamen ITD-Forschung lassen sich normtheoretisch auch als Debatten über das *meaning-in-use* begreifen. Es bestehen unterschiedliche Auffassungen darüber, welches Verhalten im Sinne einer (neuen) Norm künftig normalerweise erwartet werden kann und soll. Diese Dynamiken wirken auf unterschiedlichen Ebenen: Innerhalb der Projekte und den direkt Beteiligten, zwischen verschiedenen Projekten derselben Förderlinie (etwa in Querschnittsprogrammen), zwischen Forschenden und politisch Entscheidenden, aber schließlich auch über die Projekte hinaus. Denn durch Agenda-Setting-Prozesse, Programmkonferenzen oder Begleitforschung setzten ITD-Projekte normative Impulse oder intensivieren diese durch die (kollektive) Fortschreibung forschungspolitischer Programmlinien, bei denen die beteiligten Partner*innen und Disziplinen als Gatekeeper wirken.

Der Fokus auf Transformationswissen impliziert, dass ein Teil der erzeugten Ergebnisse in konkrete Empfehlungen für Politik und weitere Akteur*innen übersetzt werden soll. Ausgehend von einem bestimmten Problem – etwa Plastikverschmutzung – und einer normativen Haltung zum Problem – soll vermieden werden – schlagen wir als Forschende konkrete Verhaltensweisen vor. Somit werden von Forschenden Impulse für normative Dynamiken erwartet, die den Aktivitäten von Normunternehmer*innen ähneln: Beide rahmen einen normativen Sachverhalt neu, um an ihm eine aktualisierte Verhaltenserwartung festzumachen (Finnemore und Sikkink 1998). Die Beobachtungen zeigen, dass sich unsere Rolle als Forschende innerhalb des Forschungsprozesses wandelt: Einerseits sind Forschende durch ihre Beschäftigung in und mit ITD-Ausschreibungen und daraus folgenden Projekten *Normtaker* der geldgebenden Institutionen und ihrer ITD-Programme. Zugleich sind sie durch die explizite Aufforderung zu Handlungsempfehlungen für die Praxis auf anderer Ebene auch *Normmaker* und somit selbst an Normdynamiken unmittelbar wie explizit beteiligt. Schließlich entwickeln sich innerhalb der Projekte zeitweilig Positionierungen, die eher *Antipreneurtum* entsprechen, weil sie auf die Zurückweisung neuer Angemessenheitsstandards abzielen.

### Reflexion: Rollenkonflikte und Problemlösungshaltung

Die Reflexion unserer Beobachtungen greift die Erkenntnisse zu Macht und Situiertheit der kritischen IB-Normenforschung auf, die einerseits das Ausblenden von Machtbeziehungen kritisiert, andererseits die eigene Rolle von Forschenden im Forschungsprozess hinterfragt (Glaab et al. 2021). Dabei wurde die explizite Thematisierung von Macht durch interpretative, dekonstruierende und postkoloniale Ansätze in die IB-Normenforschung eingeführt. Machtblindheit manifestiert sich etwa, wenn Beziehungen zwischen Forschenden untereinander oder zu Praxisakteur*innen nicht bezüglich bestehender Hierarchien kritisch beleuchtet werden oder scheinbare Normalitäten unhinterfragt bleiben (Epstein 2014). Demgegenüber kann eine reflexive Perspektive Aspekte aufgreifen, die sich mit den Machteinschreibungen innerhalb der Praxis von Wissenschaft beschäftigen. Autobiografische, textliche und forschungsfeldspezifische Situiertheiten, machen die an bestimmte Kontexte gebundene Position von Forschenden, die normativ-politische Rolle von Daten und letztlich Wissen sowie die Bedeutung von Interaktionen im Forschungsprozess sichtbar, die sich stets aus spezifischen Quellen speisen. In diesem Zusammenhang fragt Situiertheit, welche normativen Vorstellungen eingetragen, verhandelt und konsolidiert werden (Neumann und Neumann 2015).

Die Betonung der aktiven Rolle von Forschenden für gesellschaftlichen Wandel via ITD-Forschung wirkt zunächst befremdlich, üblicherweise überlassen Forschende politisches Engagement den NGOs und anderen Lobbygruppen. Tatsächlich scheint der Unterschied zwischen Aktivisti in Lützerath oder im Hambacher Forst und uns als Forschenden in drei ITD-Nachhaltigkeitsprojekten offenkundig zu sein. Dennoch können Forschende in ITD-Projekten als Normunternehmer*innen identifiziert werden, weil ITD in einer reflexiven Perspektive die Agency der Forschenden erhöht. Die normative Festlegung über das Nachhaltigkeitsparadigma, so uneindeutig sie im Detail auch sein mag, sowie die Umsetzungsperspektive der ITD ermöglichen es Forschenden eher, die Rolle politischer Subjekte einzunehmen als das für Forschende der Fall ist, die ausschließlich im Hochschulkontext arbeiten. Durch eine breitere Reichweite und zusätzlichen Erkenntnisgewinn kann die Agency von Forschenden gestärkt werden, wenn Forschung in praktische Anwendung gebracht und durch eine teils nichtakademische, auf jeden Fall jedoch heterogene, außeruniversitäre Hörer*innenschaft rezipiert und kommentiert wird.

Schließlich verweisen unsere Beobachtungen auf Hierarchien hinsichtlich der Zuschreibung von Expertise in zweierlei Hinsicht: Erstens wird der Politikwissenschaft häufig eine Expertise bei als *weicher* wahrgenommenen Themen zugeschrieben und diese auch spezifisch nachgefragt. Dazu gehört der breite Bereich der Governance, Steuerungs- und Gerechtigkeitsfragen im Nachhaltigkeitsparadigma einschließlich Geschlechtergerechtigkeit, vermehrt Diversität und Inklusion, aber auch gesellschaftliche Beteiligung und Akzeptanz. Allerdings ist diesen Themen gemein, dass sie sich weniger gut in die den ITD-Projekten eigene Verwertungslogik einfügen lassen, sind sie doch eher reflexiv und häufig kritisch hinterfragend auf den eigenen Gegenstand bezogen als outputorientiert. Demgegenüber scheint es Forschung im rationalistischen Paradigma leichter zu haben, einschließlich jener in der Politikwissenschaft; auch weil – so die stereotype Zuspitzung – Messen, Zählen und Wiegen sich in einer medialisierten Öffentlichkeit, beim Reporting gegenüber Mittelgebenden oder in der Beratung von politisch Entscheidenden besser kommunizieren lassen als etwa die abwägende Diskussion von strukturellen Bedingungen für und Perzeptionen von Umweltgerechtigkeit. Damit verbunden ist zweitens unsere Beobachtung, dass die dem Nachhaltigkeitsparadigma innewohnende Dringlichkeit – etwa durch die Berichterstattung des Weltklimarats oder aber auch das verstärkte Erleben von Extremwetterereignissen durch die Bevölkerung – eine Forschung bevorzugt, der ein Rekurs auf die eigene Objektivität und Neutralität selbstverständlich ist. Forschung, die auf Graustufen oder Uneindeutigkeiten verweist, wird angesichts gesellschaftlicher Katastrophen und häufig auch angesichts fehlender öffentlicher Akzeptanz für Nachhaltigkeitspolitiken als eher die Komplexität der Problemlösung noch erhöhend wahrgenommen.

Beide Aspekte legen nahe, dass die ITD eine spezifische Art des Forschens strukturell begünstigt. Dadurch werden bestimmte Akteur*innen in eine priorisierte Position versetzt und gleichzeitig dazu befähigt, über andere Akteur*innen und disziplinäre Perspektiven Macht auszuüben – sei es disziplinierend oder ermöglichend. Die Zuschreibung von Expertise in der ITD-Nachhaltigkeitsforschung kann also angesichts der Hinwendung zur Öffentlichkeit (Beratung, Wissenschaftskommunikation) und Dringlichkeit (zur nachhaltigen Entwicklung) auch bestehende Hierarchien als offensichtliche oder verdeckte Machtunterschiede konsolidieren, ohne dass diese im Konsortium explizit gerechtfertigt oder anderweitig legitimiert werden müssten. Schließlich wirken sich die verschiedenen Anforderungen an Forschende in ITD-Kontexten auch auf die Situiertheit aus, indem sie Rollenkonflikte initiieren und die Grenzen zwischen Wissenschaft und politischer Betätigung verwischen oder infrage stellen.

## Schlussfolgerungen und Implikationen der ITD als normativer Dynamik für die Politikwissenschaft

Wir haben in diesem Beitrag mithilfe der IB-Normenforschung die Verbreitung der ITD in der Politikwissenschaft beschrieben und erörtern im Folgenden auf Grundlage unser Nachhaltigkeitsprojekte die Implikationen für Forschende und Forschung. In einem ko-konstitutiven Verständnis von Akteur*in und Struktur werden normative Dynamiken durch Handlungen von Akteur*innen sowie durch die Beharrungskräfte normativer Ordnungen ermöglicht und beschränkt, wodurch sich eine reflexive Perspektive ergibt, die Normen als stabil, aber auch wandelbar denkt. Normative Dynamiken lassen sich somit nicht im Hinblick auf einzelne Phänomene und Akteur*innen isolieren, vielmehr sind sie multidirektional und beziehen sich bei der ITD zunächst auf die Art und Weise wie Forschung gemacht wird, welche Erwartungen an sie gestellt werden und wie sich Forschende verhalten sollen. Zugleich wirkt das daraus resultierende Verständnis von Forschung und ihren Akteur*innen auf ITD zurück und schreibt die Dynamik fort. ITD adressiert allerdings auch gesellschaftliche Normen jenseits von Forschungsnormen (u. a. Nachhaltigkeit oder Partizipation), wirkt in unterschiedliche Richtungen (innerhalb und außerhalb spezifischer ITD-Projekte) und tangiert verschiedene Akteur*innen (u. a. Fördermittelgebende, Forschende, politische und gesellschaftliche Akteur*innen). Wie wir anhand des bereits bekannten Spektrums zwischen Wissensintegration, Beteiligung und Anwendungsbezügen verdeutlichen, eröffnet die ITD für die Politikwissenschaft gleichermaßen Chancen und Herausforderungen. Dabei ist zu beachten, dass die Implikationen je nach Verortung eines konkreten Forschungsprojekts auf dem Spektrum variieren können. Insgesamt aber stellt die ITD sowohl disziplinäres als auch gesamtgesellschaftliches Veränderungspotenzial bereit, für das es gilt, die politikwissenschaftliche Expertise in sowie über ITD zukünftig noch weiter auszuschöpfen und die Implikationen für die Disziplin und deren Forschung intensiver zu reflektieren. Dazu weisen wir im Folgenden weitere Perspektiven jenseits der IB-Normenforschung aus, die unsere empirischen Ergebnisse ergänzen können.

Durch die in der ITD-Forschung umgesetzte *Wissensintegration *wird die kollaborative Bearbeitung komplexer gesellschaftlicher Probleme möglich, wobei Verständigung und Dialog disziplinäre und universitäre Grenzen überschreiten sollen. Dies kann positive Effekte für Politikwissenschaftler*innen und deren Forschung mit sich bringen, wenn der fremde Blick auf die eigene Fachkultur die Reflexionsfähigkeit von Forschenden erhöht und eine ganzheitliche Problembetrachtung ermöglicht. Gleichzeitig ergibt sich für die Politikwissenschaft in der ITD-Forschung ein Zugewinn an Gestaltungsmacht, weil die Sichtbarkeit der Disziplin potenziell gesteigert wird. Im Sinne einer Ermöglichung verschafft ITD-Forschung der Politikwissenschaft einen Platz am Tisch der großen gesellschaftlichen Fragen, den diese für sich und auch in Konkurrenz zu anderen Disziplinen (und Stakeholdern wie forschenden Unternehmen) produktiv nutzen kann: Die damit verbundene Einwerbung von Drittmitteln sowie die potenziellen Effekte für Netzwerkbildung, Wissenskommunikation und die Bedeutung einer als kompetent wahrgenommen und öffentlich präsenten Politikwissenschaft können als positiv gewertet werden. Unterstellen wir damit gleichsam eine politisch aktive Disziplin, erhält die Politikwissenschaft auch die Autorität, ein anderes Nachdenken beispielsweise über Mensch-Umwelt-Beziehungen in den Mainstream und die politische Öffentlichkeit zu tragen; womöglich auch im Kontrast zu Debatten, die naturwissenschaftlich-technischen Paradigmen nahe zu stehen scheinen.

Dieser potenzielle Zugewinn an Bedeutung und die damit verbundene Macht zur Gestaltung verweisen auf die Relevanz der universitären Politikwissenschaft in der sogenannten Expertokratie. ITD katalysiert laufende Debatten über die Selbstverortung sowie Selbstvergewisserung der Politikwissenschaft (Schöne und Bergem 2022) und rekurriert auf innovative Formen des Wissensaustauschs mit Politik und Gesellschaft (Hegemann und Niemann 2022). Dies ist nicht nur für die Politikwissenschaft relevant, sondern erstreckt sich auch auf das Verhältnis zu anderen Disziplinen, bei denen die Beteiligung an Normsetzungsprozessen zur gängigen Praxis gehört – etwa in der Rechtswissenschaft bei ISO-Normierungen, für die Chemiker*innen der Europäischen Chemikalienagentur oder in der klassischen Politikberatung durch Stiftungen und Thinktanks. Insofern kann die ITD auch als Chance gesehen werden, wissenschaftliche Expertise in den gesellschaftlichen Prozess einzubringen.

Zugleich steht einer Steigerung der Agency von Forschenden eine potenzielle Bevormundung, möglicherweise sogar eine Beschränkung wissenschaftlicher Freiheit gegenüber. Indem über die Verbreitung von ITD bestimmte Formen des Forschens – über Disziplinen und Akteur*innengruppen hinweg – zu einem Angemessenheitsstandard erhoben werden, der die Güte von Forschung beeinflusst, sind Themen, Theorien und Methoden zwar immer noch frei wählbar, gleichzeitig drohen durch die Entscheidung dafür womöglich Verluste von Fördermitteln und Anerkennung. Ein Verzicht auf Drittmittelprojekte mit größeren ITD-Anteilen samt ihrer Mittel für Mitarbeitende, Feldforschung und (Open-Access‑)Publikationen erscheint angesichts spärlich ausgestatteter Hochschulen nicht im Einklang mit den Kriterien für wissenschaftlichen Erfolg zu stehen.

Fragen danach, was an Forschung angemessen ist und welches Konfliktpotenzial sie birgt, zeigen sich auch im Hinblick auf Wissen. ITD kann eine spezifische Art des Forschens strukturell begünstigen und somit eine Bevorzugung bestimmten, nämlich technologisch-wissenschaftlichen Wissens, zur Folge haben. Es besteht das Risiko einer Reproduktion von Wissenshierarchien, wenn zwischen Disziplinen und deren favorisierten Forschungsparadigmen oder zwischen Expert*innen und Laien gerade jene Wissensbestände selektiert werden, die im Einklang mit dem stehen, was an Output erwünscht wird. Durch diese vorauseilende Disziplinierung von Forschungsoptionen kann ITD ein rationalistisches Forschungsparadigma (auch in der Politikwissenschaft) perpetuieren und birgt die Gefahr der Marginalisierung kritisch-emanzipatorischer heterodoxer Forschungsansätze. Dies schließt nicht nur, aber insbesondere soziokulturelles Wissen in der Forschung über Nachhaltigkeit mit ein. Diese Überlegungen fortführend könnten zum Beispiel diskurstheoretische Untersuchungen in genealogischer Perspektive klären, wie es zu bestimmten Vorstellungen von Wissensintegration und deren Zielsetzungen gekommen ist, wo Auslassungen bestehen und inwiefern sie (Forschungs‑)Subjekte empowern oder disziplinieren. Zudem ließe sich wissenssoziologisch fragen, ob Wissensintegration im Sinne der ITD zu mehr Stabilität oder zu mehr Wandel in Forschungslandschaft und Wissen(schaft)spolitik führt und unter welchen Bedingungen sie zu einem Aufbrechen tradierter Strukturen führen könnte.

Politikwissenschaft und Gesellschaft können vom *Anwendungsbezug* der ITD profitieren und die Ziele gelingender ITD in den Förderlinien von drittmittelgebenden Institutionen lesen sich sehr vielversprechend: Soziale und technologische Innovation und Problemlösung sollen zu breiter Akzeptanz, beschleunigtem Wandel und gesteigerter Effektivität von Policies führen. Allerdings kann der Anwendungsbezug der ITD für eine Überbetonung des Forschungsoutputs sorgen. In der Absicht, akzeptier- und umsetzbares Wissen für komplexe Problemzusammenhänge zu generieren, welches disziplinäre Vorschläge allein nicht mehr bereitstellen können (oder wollen), kulminieren in der Wissensintegration zentrale Ansprüche an ITD-Ergebnisse, nämlich bezüglich ihrer Effektivität im Sinne der Problemlösung und ihrer Legitimität als Akzeptanzbeschaffung. Die in der Analyse angesprochene Diskussion zur Machbarkeit von ITD-Projekten strukturiert den angestrebten Erkenntnisfortschritt mit. Die Internationale Politische Ökonomie könnte hier beispielsweise analysieren, ob und inwiefern deliberative Energie – von Dissidenz ganz zu schweigen –, zivilgesellschaftliches Engagement sowie Forschung hier marktähnlichen Effizienzlogiken angepasst werden.

Obwohl die sogenannte Auftragsforschung diese Problematik kennt und sie nicht ausschließlich der ITD zu eigen ist, birgt die Problemlösungs- und Anwendungsorientierung von ITD-Forschung potenzielle Rollenkonflikte für Politikwissenschaftler*innen, wenn diese zu Normunternehmer*innen werden. Zwar kann auch universitäre monodisziplinäre Forschung normative Sachverhalte neu rahmen, doch Politikwissenschaftler*innen in ITD-Forschung werden dazu angehalten, Wissensbestände in möglichst eindeutige Empfehlungen für die Praxis zu überführen und gesellschaftliche Normen unmittelbar zu setzen oder zu initiieren. Die Dissemination von Projektergebnissen durch Transfer und Wissenschaftskommunikation ist häufig in den jeweiligen Vorhaben festgeschrieben. Ergebnisse sollen aus dem Projekt heraus in die Gesellschaft, aber auch über Projektträger zu den jeweiligen Ministerien kommuniziert werden. In vielen Programmlinien können spezifische Newsletter, Programmkonferenzen und praxisnahe Symposien für die Kommunikation von Ergebnissen genutzt werden. Mit dem Transfer von Wissen erfolgt gleichzeitig eine gesellschaftliche Positionierung, die auch als Normimplementation oder zumindest deren Versuch gelesen werden kann. Hier zeigt sich die Notwendigkeit zur kritischen-reflexiven Beleuchtung der Situiertheit von Politikwissenschaftler*innen in auf Kommunikation, Anwendung und verwertbare Lösungen abzielender ITD-Forschung. Sie ermöglicht den eigenen (wissenschaftlichen) Beitrag zum gesellschaftlichen Fortschritt, die Situiertheit des Feldes sowie die der Daten zu reflektieren und zu benennen (Neumann und Neumann 2015).

Die ITD hat längst die Grenzen der Forschung im engeren Sinne verlassen und ist als Teilhabepostulat insbesondere in der umweltpolitischen Praxis angekommen (Esguerra und van der Hel 2021). Dabei setzt sie als Chance für die Politikwissenschaft nicht nur forschungspraktische oder konzeptionelle Effekte frei, sondern erzeugt durch die größere Nähe zur Öffentlichkeit und das Zusammenwirken von Forschung und Praxis auch politische Effekte. Die ITD berührt durch ihr Augenmerk auf *breite Beteiligung* die normative wie praktische Ermöglichung von Partizipation und Diversität. Somit stellt ITD ein Mehr an Beteiligungsräumen bereit, in denen eine aktive Auseinandersetzung von Politikwissenschaftler*innen mit anderen Disziplinen und Praxisakteur*innen möglich wird und sie unterschiedliche Angemessenheitsstandards verhandeln. Selbst wenn divergierende Positionen und Wertvorstellungen dabei nicht aktiv aufgelöst werden (sollen), kann die Heterogenität der Perspektiven über die in der ITD angelegte Institutionalisierung von Beteiligung und Austausch produktiv werden. ITD kann hier zu einem Empowerment von Akteur*innen beitragen, indem spezifische, lokalen Kontextbedingungen Rechnung tragende Formate zu einer politischen Aktivierung oder gar Mobilisierung beitragen und das Verständnis von Problemlagen und Handlungsperspektiven mehren. Hier birgt ITD Potenziale für die Steigerung der Folgebereitschaft von Betroffenen sowie für die Legitimität von politischen Prozessen und Ergebnissen bis hin zu Policies. In diesem Sinne setzt ITD als normative Dynamik Impulse für die Akzeptanz der an ihr beteiligten Akteur*innen, sodass etwa eine Identifikation mit nachhaltiger Entwicklung angestoßen werden kann. Dies bereitet prinzipiell auch deliberativen Beteiligungsoptionen in Richtung *ownership*, sozialem Lernen oder *environmental citizenship* den Boden, die demokratisierende Effekte haben können.

Allerdings wird die Teilhabeforderung in ITD-Projekten unterschiedlich umgesetzt und auch die Kriterien für gelingende Partizipation oder Ko-Kreation von Wissen variieren. Das Potenzial für Partizipation in der ITD lässt sich nur unter bestimmten Bedingungen (institutionell und diskursiv) realisieren und diese stellen eine Herausforderung dar. Dazu zählt zunächst, dass in Beteiligungsprozessen nicht nur bestehende Diskurse und Normen bedient werden, sondern eine prinzipielle Offenheit für neue Perspektiven und Ansätze vorhanden sein muss. Hier zeigt sich die normalisierende Macht von Fördermittelgebenden und Projektbeteiligten innerhalb der ITD, die zwar wichtige Nachhaltigkeitsthemen überhaupt erst pushen und für Partizipation öffnen können, gleichzeitig durch *invited spaces* und Agendasetzung eine inhaltliche Themenauswahl vorgeben. Hinzu kommt die Organisation von Partizipationsprozessen selbst, die vom Fragebogen über vielfältige Dialogformen bis hin zu institutionell und finanziell abgesicherten sowie und auf Dauer gestellten Formaten reicht.

Wichtig ist zudem die Auswahl der am ITD-Forschungsprozess beteiligten Akteur*innen, die sich im Hinblick auf die Repräsentativität nicht auf die quasi berufsmäßig Engagierten der Verbände und die sogenannten üblichen Verdächtigen beschränken sollte, da sonst die Gefahr droht, bestehende Asymmetrien in der Beteiligung zu perpetuieren und strukturell wiederum diejenigen zu begünstigen, deren Bedürfnisse auch ohne direkte Beteiligung Teil des Diskurses sind. Die Verbreitung von ITD und die sich verändernde Situation forschender (und damit zugleich politisch handelnder) Akteur*innen, beispielsweise als Gatekeeper in Beteiligungsprozessen, verlangen also, Fragen der Repräsentation von Interessen innerhalb von Forschung sowie deren demokratischer Relevanz zu thematisieren. Außerhalb der Forschung kann überdies danach gefragt werden, welche Forschung Gehör findet und welche Kriterien zur Aufnahme in den politischen Prozess führen.

Diese Herausforderungen berühren eine der Kernkompetenzen der Politikwissenschaft, nämlich die Erforschung von Bedingungen für offene, gleichberechtigte, faire und demokratische Teilhabe und Modi der legitimen und effektiven Politikgestaltung. Aus demokratietheoretischer Perspektive sind die in ITD-Projekten etablierten Legitimationsgründe, Verfahren und darauf basierenden Politikempfehlungen kritisch zu spiegeln. Nicht allein, weil mit ITD Gemeinwohlziele ja auch verfehlt werden können, sondern auch, weil Reallabore zumindest inkrementellen Wandel zum Ziel haben, obschon sie weder auf Mehrheitsentscheidungen basieren noch die Bedingungen eines Labors gänzlich hinter sich lassen können. Es stellt sich daher die Frage ob, und falls ja, inwiefern sich Beteiligungsprozesse und handelnde Forscher*innen in ITD-Projekten an denselben demokratietheoretischen Postulaten von Responsivität, Selbstbestimmung und Rechenschaftspflichtigkeit als Herrschaftskontrolle messen lassen müssen wie die autoritative staatliche Regulierung selbst (Schwindenhammer 2020). Zu erörtern wären anschließend an Flohr et al. (2010) weitere Fragen: Inwiefern werden in ITD-Projekten allgemein anerkannte Probleme aufgegriffen und gesellschaftlich akzeptierte Zielsetzungen verfolgt (Responsivitätskriterium)? Wird die Mitwirkung unterschiedlicher Anspruchsgruppen gesichert (Beteiligungsverfahren und Regeln) und der Willen auch marginalisierter sozialer Gruppen ermittelt und beachtet (Kriterium der Selbstbestimmung)? Ist sichergestellt, dass der Input von Wissenschaftler*innen nicht ungeprüft in den Normenbestand übernommen wird (Rechenschaftspflichtigkeit), beispielsweise durch Transparenz‑, Überwachungs- und Kontrollmechanismen (*peer-feedback*, professionelle und reputationsbasierte Verantwortung)? Selbst wenn diese Bedingungen erfüllt und ein Reallabor oder eine Stakeholderdiskussion die Partizipationspotenziale von ITD bestmöglich ausschöpft, können diese Formen der Beteiligung in der ITD den breiten gesellschaftspolitischen Diskurs über drängende Fragen, auch und gerade in Zeiten der Krise, zwar ergänzen, aber kaum substituieren.

Die wissenschaftlich und gesellschaftlich wichtigen Fragen, die aus demokratietheoretischer Sicht an die ITD gerichtet werden können, bilden ein interessantes Spannungsfeld mit dem Anspruch der ITD zur Wissensintegration; insbesondere im Hinblick auf Transformations- und Orientierungswissen. Die Nachhaltigkeitsforschung im ITD-Bereich steht selten im Verdacht, die Orientierung am Gemeinwohl zu verfehlen. Allerdings ist diese Forschung gerade mit der Hinwendung zur Öffentlichkeit mit sehr unterschiedlichen Vorstellungen darüber konfrontiert, wie tiefgreifend die Transformation im Nachhaltigkeitsbereich letztendlich ausfallen soll. Die verbreitete Haltung in der ITD, dichotome Konstruktionen von Expert*innen- und Laienwissen, Universität und Gesellschaft oder Kultur und Natur mittels Beteiligungsverfahren und Transformation aufzubrechen, macht die Kontestation der Normen in der ITD selbst sichtbar. Genau an dieser Stelle kann die Politikwissenschaft durch die Reflexion von ITD viel Forschungspotenzial heben und weitere fachliche Perspektiven können unsere empirischen Ergebnisse ergänzen. Die Forschung zu sozialen Bewegungen kann hier thematisieren, ob und wie gesellschaftliche Mobilisierung und Protest die ITD beeinflussen und vice versa. Zudem könnte die Zivilgesellschaftsforschung die Bedingungen und Mechanismen adressieren, wie ITD-Forschung über die gelingende Einbeziehung vulnerabler Gruppen in Partizipationsprozessen zur Steigerung von Legitimität beitragen kann. Populismusforschung kann auf die Stellen blicken, wo Klimawandelskeptizismus und Verschwörungstheorie durch ITD-Projekte eine Bühne erhalten und ob und wenn ja, wie diese zur Wissenschaft, aber auch zu Anti-Feminismus, Rassismus und Klassismus sprechen. Mit anderen Worten: Die sich verbreitende ITD verweist auf vielfältige politikwissenschaftliche Forschungs- und Reflexionsperspektiven, für die wir mit diesem Beitrag einen Anstoß geben möchten.

## References

[CR1] Acharya A (2004). How ideas spread: Whose norms matter? Norm localization and institutional change in Asian regionalism. International Organization.

[CR2] Adams TE, Ellis C, Bochner AP, Ploder A, Stadlbauer J, Mey G, Mruck K (2020). Autoethnografie. Handbuch Qualitative Forschung in der Psychologie.

[CR3] Ansell CK, Torfing J (2021). Public governance as co-creation. A strategy for revitalizing the public sector and rejuvenating democracy.

[CR4] Axelrod R (1986). An evolutionary approach to norms. American Political Science Review.

[CR5] Becker E (2002). Transformations of social and ecological issues into transdisciplinary research.

[CR6] Beer B, Diaz-Bone R, Weischer C (2015). Beobachtung, teilnehmende. Methoden-Lexikon für die Sozialwissenschaften.

[CR7] Betts A, Orchard P, Betts A, Orchard P (2014). Introduction: The normative institutionalization-implementation gap. Implementation and world politics: How international norms change practice.

[CR8] Birkholz S, Bochmann A, Schank J, Birkholz S, Bochmann A (2020). Ethnografie und Teilnehmende Beobachtung. Handbuch Methoden der Politikwissenschaft.

[CR9] Bloomfield A (2016). Norm antipreneurs and theorising resistance to normative change. Review of International Studies.

[CR14] BMBF. 2015. *Sozial-ökologische Forschung. Förderkonzept für eine gesellschaftsbezogene Nachhaltigkeitsforschung 2015–2020. *Bonn. https://www.fona.de/medien/pdf/pdf_8rch1v/SOEF_Foerderkonzept_barrierefrei.pdf?m=1576758321&. Zugegriffen: 13. Juli 2021.

[CR12] BMBF. 2016a. Richtlinie zur Förderung von Forschungsvorhaben der Agrarforschung unter dem Namen „Agrarsysteme der Zukunft“ im Rahmen der „Nationalen Forschungsstrategie BioÖkonomie 2030“. Bundesanzeiger vom 10.08.2016. https://www.bmbf.de/foerderungen/bekanntmachung-1231.html. Zugegriffen: 17. Juli 2021.

[CR13] BMBF. 2016b. Richtlinien zur Förderung von Vorhaben zu „Plastik in der Umwelt – Quellen, Senken, Lösungsansätze“. Bundesanzeiger vom 10.06.2016. https://www.bmbf.de/foerderungen/bekanntmachung-1195.html. Zugegriffen: 17. Juli 2021.

[CR10] BMBF. 2020a. Forschung für Nachhaltigkeit. Eine Strategie des Bundesministeriums für Bildung und Forschung. https://www.bmbf.de/SharedDocs/Publikationen/de/bmbf/7/31638_Forschung_fuer_Nachhaltigkeit.pdf?__blob=publicationFile&v=5. Zugegriffen: 12. Juli 2021.

[CR11] BMBF. 2020b. Gesellschaft verstehen – Zukunft gestalten. BMBF-Rahmenprogramm für die Geistes- und Sozialwissenschaften (2019–2025). https://www.bmbf.de/SharedDocs/Publikationen/de/bmbf/4/31500_Gesellschaft_verstehen_Zukunft_gestalten.pdf?__blob=publicationFile&v=3. Zugegriffen: 12. Juli 2021.

[CR15] Bogner A (2021). Die Epistemisierung des Politischen. Wie die Macht des Wissens die Demokratie gefährdet.

[CR16] Breidenstein G, Hirschauer S, Kalthoff H, Nieswand B (2020). Ethnografie. Die Praxis der Feldforschung.

[CR17] Breitmeier H, Schwindenhammer S, Checa A, Manderbach J, Tanzer M (2021). Aligned sustainability understandings? Global inter-institutional arrangements and the implementation of SDG 2. Politics and Governance.

[CR18] Breitmeier H, Schwindenhammer S, Checa A, Manderbach J, Tanzer M (2021). Politicized sustainability and agricultural policy: comparing norm understandings of international organizations. Journal of Comparative Policy Analysis: Research and Practice.

[CR19] Brigg M, Bleiker R (2010). Autoethnographic International Relations: exploring the self as a source of knowledge. Review of International Studies.

[CR20] Brunnengräber A, Smeddinck U, Smeddinck U, Kuppler S, Chaudry S (2016). Möglichkeiten und Grenzen der Vereinheitlichung wissenschaftlicher Begriffe in der interdisziplinären Zusammenarbeit. Inter- und Transdisziplinarität bei der Entsorgung radioaktiver Reststoffe.

[CR21] Büttner S, Laux T (2021). Umstrittene Expertise. Zur Wissensproblematik der Politik.

[CR22] Chaudry S, Plischke E, Smeddinck U, Kuppler S, Chaudry S (2016). Wissenschaftliche Synthese bei der Forschung zur Entsorgung radioaktiver Reststoffe in der Forschungsplattform ENTRIA. Inter- und Transdisziplinarität bei der Entsorgung radioaktiver Reststoffe.

[CR23] Davies GR (2013). Appraising weak and strong sustainability: searching for a middle ground. Consilience.

[CR24] Deitelhoff N (2006). Überzeugung in der Politik. Grundzüge einer Diskurstheorie internationalen Regierens.

[CR25] Deitelhoff N, Zimmermann L (2019). Norms under challenge: unpacking the dynamics of norm robustness. Journal of Global Security Studies.

[CR26] DFG. 2020. Transferprojekte in Sonderforschungsbereichen. https://www.dfg.de/foerderung/programme/koordinierte_programme/sfb/antragsteller/programmelement_transfer/index.html. Zugegriffen: 12. Mai 2022.

[CR27] DFG. 2021. Förderatlas 2021: Kennzahlen zur öffentlich finanzierten Forschung in Deutschland. https://www.dfg.de/sites/foerderatlas2021/download/dfg_foerderatlas_2021.pdf. Zugegriffen: 28. Okt. 2022.

[CR28] DVPW, DGS, DGSKA, DGEKW, und DGPuK. 2022. Gemeinsame Stellungnahme der sozialwissenschaftlichen Fachverbände Deutschlands. Berlin. https://soziologie.de/fileadmin/user_upload/stellungnahmen/Gemeins._Stellungnahme_Sozialwiss._Fachverbaende_20220315.pdf. Zugegriffen: 12. Mai 2022.

[CR29] Engelkamp S, Glaab K, Renner J (2012). ‚In der Sprechstunde‘: Wie (kritische) Normenforschung ihre Stimme wiederfinden kann. Zeitschrift für Internationale Beziehungen.

[CR30] Epstein C (2012). Stop telling us how to behave: socialization or infantilization?. International Studies Perspectives.

[CR31] Epstein C (2014). The postcolonial perspective: an introduction. International Theory.

[CR32] Esguerra A, van der Hel S (2021). Participatory designs and epistemic authority in knowledge platforms for sustainability. Global Environmental Politics.

[CR33] Feldhoff B, Stockmann N, Fanderl N, Gahle A-K, Graf A, Leger M, Sonnberger M (2019). Bridging theories and practices: Boundary objects and constellation analysis as vehicles for interdisciplinary knowledge integration. Sustainability.

[CR34] Finnemore M, Sikkink K (1998). International norm dynamics and political change. International Organization.

[CR35] Flohr A, Rieth L, Schwindenhammer S, Wolf KD (2010). The role of business in global governance.

[CR36] Froese A, Woiwode H, Suckow S (2019). Mission Impossible? Neue Wege zu Interdisziplinarität. Empfehlungen für Wissenschaft, Wissenschaftspolitik und Praxis.

[CR37] Fröhlich M, Korte K-R, Schirm SA, Vorländer H (2017). Unser Fach Politikwissenschaft. Zeitschrift für Politikwissenschaft.

[CR38] Gibbons M, Limoges C, Nowotny H, Schwartzman S, Scott P, Trow M (1994). The new production of knowledge: the dynamics of science and research in contemporary societies.

[CR40] Glaab M, Bergem W, Schöne H (2022). Regierungsführung und (politik-)wissenschaftliche Politikberatung – einige Beobachtungen, Befunde und Impulse zur Diskussion. Wie relevant ist die Politikwissenschaft?: Wissenstransfer und gesellschaftliche Wirkung von Forschung und Lehre.

[CR42] Graf Antonia (2016). Diskursive Macht. Transnationale Unternehmen im Nachhaltigkeitsdiskurs.

[CR43] Graf A, Glaab K, Engelkamp S, Engelkamp S, Glaab K, Graf A (2021). Normen und andere Vehikel: Wie untersuchbar gemacht wird, was sich der Beobachtung entzieht. Kritische Normenforschung in den Internationalen Beziehungen: Neue Wege und metatheoretische Perspektiven.

[CR39] Glaab K, Engelkamp S, Engelkamp S, Glaab K, Graf A (2021). Lagerbildung und Verständigung in der kritischen Normenforschung. Eine Einleitung. Kritische Normenforschung in den Internationalen Beziehungen: Neue Wege und metatheoretische Perspektiven.

[CR41] Godemann J, Michelsen G, Bergmann M, Schramm E (2008). Transdisziplinäre Integration in der Universität. Transdisziplinäre Forschung: Integrative Forschungsprozesse verstehen und bewerten.

[CR44] Haas PM, Peterson MJ (2019). Reflections on contested knowledge and those who study it. Contesting global environmental knowledge, norms and governance.

[CR45] Halperin S, Heath O (2012). Political research. Methods and practical skills.

[CR46] Hansen-Magnusson H, Vetterlein A, Wiener A (2020). The problem of non-compliance: knowledge gaps and moments of contestation in global governance. Journal of International Relations and Development.

[CR47] Hegemann H, Niemann H, Bergem W, Schöne H (2022). Vom Transfer zur Partizipation? Die gesellschaftliche Relevanz der Friedens- und Sicherheitsforschung unter neuen Bedingungen. Wie relevant ist die Politikwissenschaft?: Wissenstransfer und gesellschaftliche Wirkung von Forschung und Lehre.

[CR48] Henkel A, Lüdtke N, Buschmann N, Hochmann L, Henkel A, Lüdtke N, Buschmann N, Hochmann L (2018). Einleitung. Reflexive Responsibilisierung.: Beiträge kulturwissenschaftlicher Perspektiven zum Nachhaltigkeitsdiskurs. Reflexive Responsibilisierung: Verantwortung für nachhaltige Entwicklung.

[CR49] Hickmann T, Lederer M, Marquardt J, Radtke J, Schwindenhammer S, Weiland S, Bergem W, Schöne H (2022). Herausforderung Nachhaltigkeitstransformation: Mehr Politikwissenschaft wagen!. Wie relevant ist die Politikwissenschaft?: Wissenstransfer und gesellschaftliche Wirkung von Forschung und Lehre.

[CR50] Hitzler R, Bohnsack R, Marotzki W, Meuser M (2006). Ethnographie. Hauptbegriffe Qualitativer Sozialforschung.

[CR51] Inayatullah N, Blaney DL (2012). The dark heart of kindness: the social construction of deflection. International Studies Perspectives.

[CR52] Jahn S, Newig J, Lang DJ, Kahle J, Bergmann M (2021). Demarcating transdisciplinary research in sustainability science: Five clusters of research modes based on evidence from 59 research projects. Sustainable Development.

[CR53] Jahn T, Bergmann M, Keil F (2012). Transdisciplinarity: between mainstreaming and marginalization. Ecological Economics.

[CR54] Jantsch E (1972). Inter- and transdisciplinary university: a systems approach to education and innovation. Higher Education.

[CR55] Jooß C, Welter F, Leisten I, Richert A, Jeschke S, Mai M (2014). Innovationsförderliches Knowledge Engineering in inter- und transdisziplinären Forschungsverbünden. Handbuch Innovationen: Interdisziplinäre Grundlagen und Anwendungsfelder.

[CR56] Jurkovich M (2020). What isn’t a norm? Redefining the conceptual boundaries of ‘norms’ in the human rights literature. International Studies Review.

[CR57] Klotz A, Lynch C (2007). *Strategies for research in constructivist international relations*. International relations in a constructed world.

[CR58] Kromrey H, Roose J, Strübing J (2016). Empirische Sozialforschung. Modelle und Methoden der standardisierten Datenerhebung und Datenauswertung mit Annotationen aus qualitativ-interpretativer Perspektive.

[CR59] Lang DJ, Wiek A, Bergmann M, Stauffacher M, Martens P, Moll P, Swilling M, Thomas CJ (2012). Transdisciplinary research in sustainability science: practice, principles, and challenges. Sustainability Science.

[CR60] Lantis JS, Sandholtz W (2017). Theories of international norm contestation: Structure and outcomes. Oxford research encyclopedia of politics.

[CR61] Lantis JS, Wunderlich C (2022). Reevaluating constructivist norm theory: a three-dimensional norms research program. International Studies Review.

[CR62] Linsenmaier T, Schmidt DR, Spandler K (2021). On the meaning(s) of norms: ambiguity and global governance in a post-hegemonic world. Review of International Studies.

[CR63] Loenhoff, Jens. 2021. Der Fluch der Interdisziplinarität. *Frankfurter Allgemeine Zeitung. *13 Mai 2021. https://www.faz.net/aktuell/karriere-hochschule/hoersaal/geisteswissenschaften-der-fluch-der-interdisziplinaritaet-17337079.html. Zugegriffen: 18. Juli 2021.

[CR64] Loges B (2013). Schutz als neue Norm in den internationalen Beziehungen. Der UN-Sicherheitsrat und die Etablierung der Responsibility to Protect.

[CR65] Loges B, Jakobi AP (2020). Not more than the sum of its parts: de-centered norm dynamics and the governance of plastics. Environmental Politics.

[CR66] Lüders C, Bohnsack R, Marotzki W, Meuser M (2006). Teilnehmende Beobachtung. Hauptbegriffe Qualitativer Sozialforschung.

[CR67] Lutz LM, Bergmann M, Holstenkamp L, Radtke J (2018). Transdisziplinarität: Forschungsansatz für die Energiewende. Handbuch Energiewende und Partizipation.

[CR68] MacKenzie M, Sesay M (2012). No amnesty from/for the International: The production and promotion of TRCs as an international norm in Sierra Leone. International Studies Perspectives.

[CR69] Michelsen G, Adomßent M, Heinrichs H, Michelsen G (2014). Nachhaltige Entwicklung: Hintergründe und Zusammenhänge. Nachhaltigkeitswissenschaften.

[CR71] Neumann IB (2010). Autobiography, ontology, autoethnology. Review of International Studies.

[CR70] Neumann C, Neumann IB (2015). Uses of the self: two ways of thinking about scholarly situatedness and method. Millennium: Journal of International Studies.

[CR72] Peterson MJ (2019). Contesting global environmental knowledge, norms, and governance.

[CR73] Renn O, Deuschle J, Jäger A, Weimer-Jehle W (2007). Leitbild Nachhaltigkeit. Eine normativ-funktionale Konzeption und ihre Umsetzung.

[CR74] Rittel HWJ, Webber MM (1973). Dilemmas in a general theory of planning. Policy Sciences.

[CR75] Rosert E, Sauer F, von Hauff L, Masala C, Springer Reference Sozialwissenschaften (2022). Normenforschung in den Internationalen Beziehungen. Handbuch Internationale Beziehungen.

[CR76] Sandholtz W, Sandholtz W (2017). International norm change. Oxford research encyclopedia of politics.

[CR77] Schneidewind U, Singer-Brodowski M (2015). Vom experimentellen Lernen zum transformativen Experimentieren – Reallabore als Katalysator für eine lernende Gesellschaft auf dem Weg zu einer Nachhaltigen Entwicklung. Zeitschrift für Wirtschafts- und Unternehmensethik.

[CR78] Schöne H, Bergem W, Bergem W, Schöne H (2022). Wie relevant ist die Politikwissenschaft? Zur Einführung. Wie relevant ist die Politikwissenschaft?: Wissenstransfer und gesellschaftliche Wirkung von Forschung und Lehre.

[CR79] Schwartz-Shea P, Yanow D (2012). Interpretive research design. Concepts and processes.

[CR80] Schwindenhammer S (2017). Global organic agriculture policy-making through standards as an organizational field: when institutional dynamics meet entrepreneurs. Journal of European Public Policy.

[CR81] Schwindenhammer S (2018). The new regionalism in global organic agricultural governance through standards: a cross-regional comparison. Global Environmental Politics.

[CR82] Schwindenhammer, Sandra. 2020. Demokratieprojekt Bioökonomie. https://www.wissenschaftsjahr.de/2020-21/aktuelles-aus-der-biooekonomie/koepfe-des-wandels/demokratieprojekt-biooekonomie. Zugegriffen: 12. Mai 2022.

[CR83] SPD, Bündnis 90/ Die Grünen, und FDP. 2021. *Koalitionsvertrag 2021–2025 zwischen der Sozialdemokratischen Partei Deutschlands (SPD), Bündnis 90 / Die Grünen und den Freien Demokraten (FDP). Mehr Fortschritt wagen. Bündnis für Freiheit, Gerechtigkeit und Nachhaltigkeit. *Berlin. https://www.spd.de/fileadmin/Dokumente/Koalitionsvertrag/Koalitionsvertrag_2021-2025.pdf. Zugegriffen: 2. Febr. 2022.

[CR84] Stockmann N, Graf Antonia (2019). Nachhaltige urbane Mobilität von morgen zwischen Reproduktion und Dekonstruktion von Normen. Amos International.

[CR85] Stockmann N, Graf Antonia (2020). “Polluting our kids’ imagination”? Exploring the power of Lego in the discourse on sustainable mobility. Sustainability: Science, Practice and Policy.

[CR86] Stockmann N, Graf Antonia (2022). Just translation? A socioecological justice lens on EU environmental governance and urban mobility transitions. Zeitschrift für Politikwissenschaft.

[CR87] Vilsmaier U, Lang DJ, Heinrichs H, Michelsen G (2014). Transdisziplinäre Forschung. Nachhaltigkeitswissenschaften.

[CR88] Vrasti W (2008). The strange case of ethnography and international relations. Millennium: Journal of International Studies.

[CR89] Vrasti W, Guillaume XPB (2017). Ethnography/autoethnography/autobiography. Routledge handbook of international political sociology.

[CR90] Wichterich, Christa. 2012. *Die Zukunft, die wir wollten. Eine Feministische Perspektive*. Schriften zur Ökologie 21. https://www.boell.de/sites/default/files/Feministische_Zukunft-i.pdf. Zugegriffen: 14. Mai 2022.

[CR91] Wiener A (2018). Contestation and constitution of norms in global international relations.

[CR92] Wissenschaftsrat. 2015. Empfehlungen zu wissenschaftlicher Integrität. Positionspapier. https://www.charite.de/fileadmin/user_upload/portal/forschung/gute-wiss-praxis/Wissenschaftsrat-Wissenschaftliche_Integrit%C3%A4t.pdf. Zugegriffen: 30. Juni 2021.

[CR93] Wissenschaftsrat (2020). Wissenschaft im Spannungsfeld von Disziplinarität und Interdisziplinarität. Positionspapier.

[CR94] Zarakol A (2014). What made the modern world hang together: socialisation or stigmatisation?. International Theory.

[CR95] Zimmermann L (2017). Global norms with a local face. Rule-of-law promotion and norm-translation.

[CR96] Zwingel S (2012). How do norms travel? Theorizing international women’s rights in transnational perspective. International Studies Quarterly.

